# Understanding the liver under heat stress with statistical learning: an integrated metabolomics and transcriptomics computational approach

**DOI:** 10.1186/s12864-019-5823-x

**Published:** 2019-06-17

**Authors:** Allen H. Hubbard, Xiaoke Zhang, Sara Jastrebski, Abhyudai Singh, Carl Schmidt

**Affiliations:** 1 Bioinformatics and Systems Biology, University of Delaware, Newark, Delaware USA; 2 Statistics, George Washington University, Washington, D.C, USA; 3 Animal and Food Sciences, University of Delaware, Newark, Delaware USA; 4 Electrical Engineering and Computer Science, University of Delaware, Newark, Delaware USA

**Keywords:** High throughput sequencing, Transcriptome, Metabolome

## Abstract

**Background:**

We present results from a computational analysis developed to integrate transcriptome and metabolomic data in order to explore the heat stress response in the liver of the modern broiler chicken. Heat stress is a significant cause of productivity loss in the poultry industry, both in terms of increased livestock morbidity and its negative influence on average feed efficiency. This study focuses on the liver because it is an important regulator of metabolism, controlling many of the physiological processes impacted by prolonged heat stress. Using statistical learning methods, we identify genes and metabolites that may regulate the heat stress response in the liver and adaptations required to acclimate to prolonged heat stress.

**Results:**

We describe how disparate systems such as sugar, lipid and amino acid metabolism, are coordinated during the heat stress response.

**Conclusions:**

Our findings provide more detailed context for genomic studies and generates hypotheses about dietary interventions that can mitigate the negative influence of heat stress on the poultry industry.

**Electronic supplementary material:**

The online version of this article (10.1186/s12864-019-5823-x) contains supplementary material, which is available to authorized users.

## Background

Obtaining biological insight from large-scale transcriptome and metabolome data is challenging due to biological and technical variance. Careful experimental design can limit unwanted noise. However, when properly harnessed, biologically driven variation can be used to prioritize signals that elude traditional enrichment analysis. For example, biological variation relating to a treatment response depends on many variables that are not easily controlled such as allelic or physiological variants. This fact can be informative because many compounds involved in the same process will have similar patterns of regulation, which can be detected as recognizable signatures in high dimensional omics data. This can be used to identify relationships between elements of the same pathway, even when their scales of expression and variance differ considerably, by relying on multi-tiered statistical learning strategies. This approach allows the combination of transcriptome and metabolome data to gain a more comprehensive biological understanding of a system. This is particularly helpful in identifying significant features from the large, complex datasets now common in dual or multi-omics studies.

The modern broiler chicken is a fundamental source of poultry meat. It has been under strong artificial selection during the past several decades for increased breast muscle yield [1]. This is thought to be at the expense of other systems, resulting in decreased heat tolerance and increased mortality during heat stress. The relationship between the altered physiology of the broiler and susceptibility to heat stress is not fully understood, however. It is believed to involve altered appetite and preferential routing of resources to muscles tissue. Such changes are systemic, influenced by both behavior and metabolism.

One organ capable of exerting strong influence on both bird growth and thermoregulation is the liver. This organ has recently proved effective as a subject for studies that leverage multi-omics approaches including transcriptomics and metabolomics [2]. Such work has shed light on differentially regulated genes and metabolites. However, a systems level understanding in which fluxes in metabolites are related to gene expression, are lacking. This is partly because computational approaches exploring the totality of a biological response including gene expression and metabolite production is lacking. We combine RNA-seq (Ribonucleic Acid Sequencing) expression and metabolites from the liver to identify genes and compounds that function as biomolecules associated with heat stress. While metabolomics data identifies changes in biologically active compounds, RNA-Seq data identifies genes that regulate metabolic changes. We offer a geometric interpretation for our statistical pipeline, composed of k-means, random forest and hierarchical clustering, describing how each algorithm contributes to a pipeline that recapitulates novel biology.

Our analysis applies statistical learning approaches on metabolite and gene expression data, restricting transcriptome analysis to a core module of liver enriched genes. These are determined by a definition we propose that proves more stringent than other types of relative expression analysis. Sub-setting in this fashion isolates tissue-enriched genes that reflect unique biology specific to the liver in a tissue diverse dataset, across a number of bird lines. The approach of sub-setting by tissue enriched genes and focusing on classifying power and clustering patterns when combined with metabolite measurements provides a framework to integrate metabolite and transcriptome data. This approach of combining data from different high-throughput technologies makes it possible to identify important features of the high dimensional dataset.

Finally, extending the work of earlier GWA (genome wide association) studies that sought to model ratios of metabolites as functions of SNP’s, (single nucleotide polymorphisms) we model metabolite ratios in terms of other metabolites. The original purpose of these GWA metabolite studies was to detect the genetic basis of metabolic changes [3]. However, modeling ratios as function of metabolites allows detection of metabolic forks, or small network motifs where precursors are selectively routed to different metabolic fates under heat stress. The compounds used to compose triplets representing possible metabolic forks are selected from hypotheses developed through the combined k-means [4] random forest [5] and a hierarchical clustering pipeline [6]. A triplet is defined as a function of the form $\mathrm{cor}\left(\mathrm{A},\frac{\mathrm{B}}{\mathrm{C}}\right)$ where A, B and C are any combination of metabolites. Candidates for A, B and C were chosen from amino acids known to be catabolized under heat stress [2] and sugar and fat molecules that may incorporate these molecules, and which are prioritized by our pipeline.

The combination of RNA-Seq with metabolite data identifies novel shifts in gene regulation that reflect pathway changes influencing metabolite levels.

Our combined informatics strategy identifies elements under biological regulation and which could be targets for selective breeding. Additionally, the identification of heat stress responsive metabolites produces candidates for feed supplementation studies.

## Methods

The heat stress response is multi-tiered and involves input from multiple tissues. At the cellular level, the heat stress response unfolds across an intricate program of organelle specific changes. Which changes are causal, and which merely correlative with underlying signal or sensing pathways, thus becomes a complex question. However, the variability associated with most basal regulators of the heat stress response should be most closely related to the variation in the downstreamm heat stress response. By the transitive nature of biological communication, the introduction of noise into the signal diminishes the capacity of downstream molecules, which correlate with, but do not cause the heat stress response, to discriminate between treatment and control samples. From this perspective, the problem of identifying causal molecules from expression profile is well posed as a statistical learning problem that can be addressed through random forests. Random forests can rank candidates on their ability to correctly identify the class of samples as assigned to control or experimental treatment groups. Our approach follows sorting compounds into initially crude clusters using k-means clustering, prior to application of the random forest algorithm. Finally, these top biomolecules are related to one another using hierarchical clustering. Genes and metabolites were standardized by z-score in order to prevent differences in the scale of data from skewing the results. All genes submitted to the pipeline had been previously sub-setted as liver enriched across a tissue-diverse dataset.

### Subsetting of transcriptome data

Liver enriched genes were defined as those genes whose z-score as calculated in the formula $\frac{\overline{x\Big(} tissue\ interest\Big)-\mu (background)}{\sigma (background)}$, is greater than 5. The background samples were acquired from a tissue diverse chicken dataset of 799 libraries utilized over a range of experiments in the various lines of chicken studied by the lab. Only genes which passed this z-score test for initial enrichment in the liver were admitted to the downstream statistical learning pipelines. Ultimately, focusing on liver enriched genes reduced the set of transcripts being utilized for downstream analyses from ~ 26,000 to 347 (Additional file 4).

**Fig. 1:**
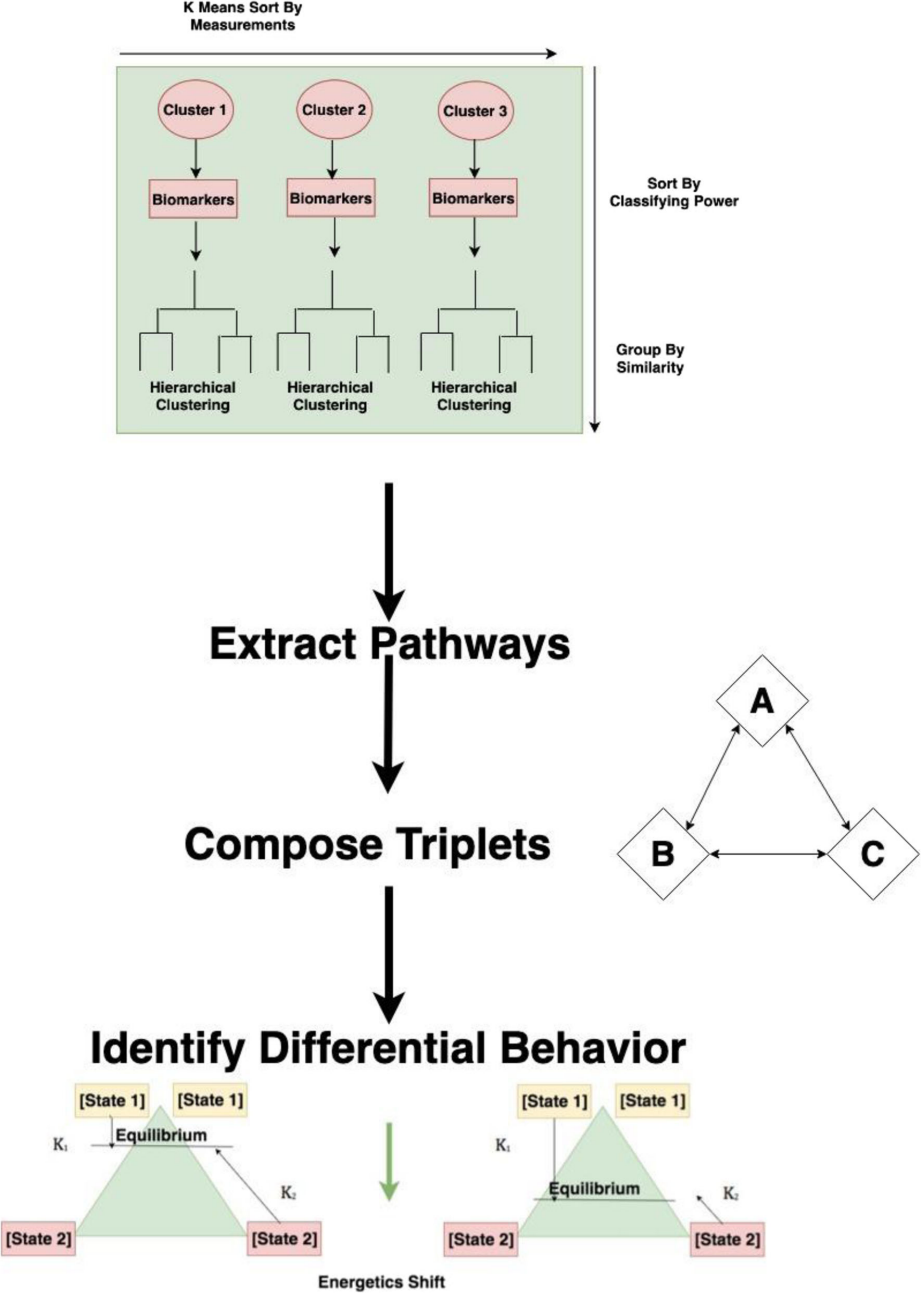
Total pipeline, from data analysis to identifying hypothetical mechanisms

Biomolecules were identified and prioritized to extract pathways from whose elements triplets could be calculated. (Fig. 1) Triplets showing differential behavior selected, which demonstrate equilibrium shifts at state assumptions and thus indicate behavior of a metabolic fork.

### Geometric and biological consideration of K-means step

**Fig. 2:**
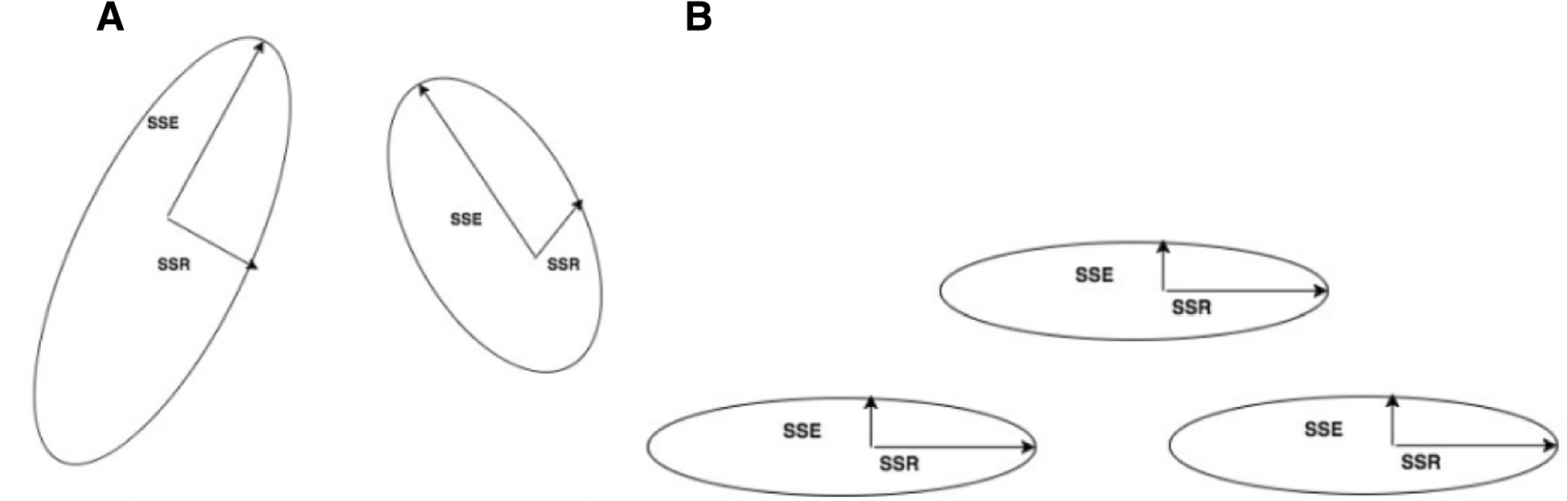
**a** and **b** Example of possible models around specific cluster with different k-means selection, illustrating more uniform clustering results with k = 3 (2B) compared to k = 2 (2A)

A goal of first leveraging k-means analysis was to build more biologically interpretable random forests, with compounds initially separated by expression patterns. This reflects the idea that pathways involving essential biological compounds occur across a spectrum of expression profiles, but may crowd one another out in downstream analyses. First grouping compounds by k-means prevented compounds from one expression profile crowding out those demonstrating another pattern, especially when they possessed similar capacities for classifying samples as control or heat stress during random forest analysis. Thus, the optimal partitioning, for this purpose, should produce clusters that are similar in explanatory power. Selecting k = 3 accomplishes this goal by distributing compounds across clusters that are as similar to one another as possible in terms of their explanatory power (Fig. 2a and b).

### Metabolic forks

Metabolic forks, in which ratio of metabolites represent activities of competing biological processes are an adaptation of concepts introduced by Gieger *et. al*, in which ratio of metabolites represent biological activity of processes influence by genotype. We refer to these regulatory triplets as such, because they represent divergent fates for metabolites. Candidates for components of metabolic forks were determined via prior knowledge as compounds established in the broiler heat stress response through r previous work [2] and which were biomolecules prioritized by the statistical learning components of the pipeline or known to be related to these biomolecules.

**Fig. 3:**
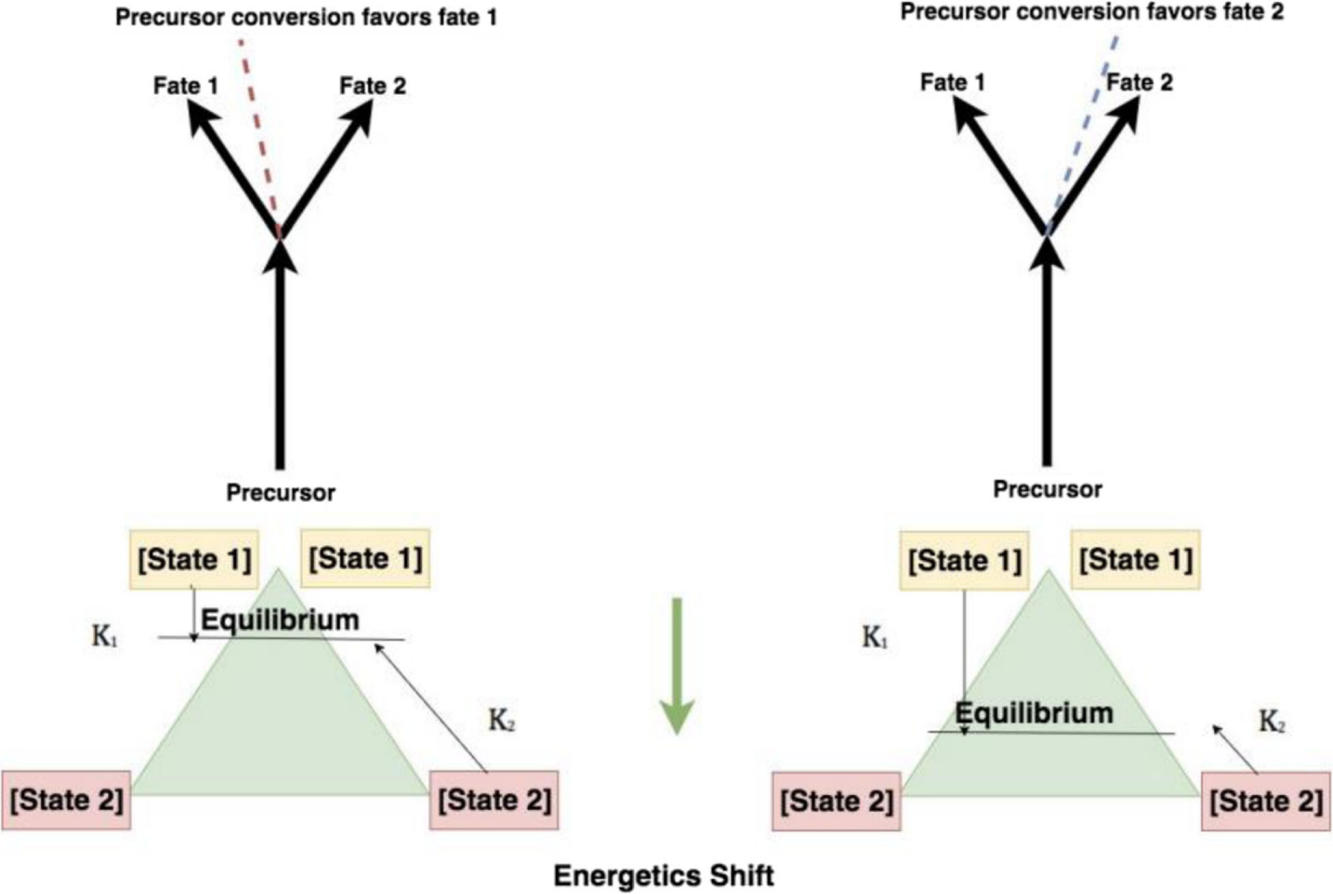
Under changes in gene expression that alter levels of the regulating enzymes, precursors are preferentially routed to one metabolic fate over another. Shifts in the ratio between metabolites representing fate 1 or fate 2 may represent shifts in biology

Such functions, relying on ratios, serve as a more realistic description of the biochemistry of pathway steps than simple correlations with raw measurements. For example, in pathway reactions where one enzyme regulates the forward reaction and another the reverse, the regulation through gene expression can cause relative increases in the product metabolite compared to the precursor metabolite. This shifts the favorability of the pathway step towards either the products or reactants. Similarly, a shift in favorability of a precursor towards one metabolic fate, at the expense of another, under regulation thus represents a “metabolic fork” (Fig. 3). Having hypothesized that amino acids from catabolized proteins fuel production of sugar and fats by providing carbon backbones, we calculated “metabolic forks” that included lipids, sugar and amino acids prioritized by the statistical learning pipeline. *P*-values were determined from the interaction term of the resulting linear model of the metabolic fork, in order to identify a significant difference in the slope between control and experimental conditions. Among metabolic forks with a significant interaction *p*-value, one was identified which represents the intersection of lipid, sugar and amino acid metabolism.

### Bird and tissue handling

Male broiler chickens *(Gallus gallus)* were obtained from Mountaire hatchery (Millsboro, DE) on day of hatch and divided into thermoneutral and experimental houses on the University of Delaware farm. This protocol has been previously described in Jastrebski et al., [2] and Hubbard et al., [7]. As described in these studies, birds were raised under a light cycle of 23 h of light and 1 h of dark. Standard management and husbandry procedures were followed, as approved by the Animal Care and Use Committee (AACUC #(27) 03–12-14R). Birds were given ad libitum access to water and fed the same diet (corn-soy) which met all NRC requirements [8]. Both groups were raised at 35 °C until one-week post hatch. Temperature was decreased 5 °C each week thereafter until temperature reached 25 °C at day 21 post hatch. The thermoneutral house was then maintained at 25 °C and the heat stress house was subject to 35–37 °C for 8 h per day, to mimic an environmental heat wave. Birds were kept in houses with sawdust bedding during the experiment including during the heat stress treatment. Eight individuals were collected for control as well as experimental treatments. Average mass at time of necropsy was 1.453 kg for heat stressed birds, while mass of control birds was 1.711 kg for control birds. Temperature in both houses was maintained by a computerized system controlling heaters and ventilation fans (Chore-time Equipment, Milford, Indiana). Temperature ranged between 35 and 37 °C during the 8 hours of heat stress. This yields an internal body temperature (cloacal) of 43.5 °C within 2 hours of the onset of heat stress. This body temperature can induce a heat stress response in chicken cells [9]. In the control (thermoneutral) house the temperature ranged between 23 and 25 °C during this same period. Both houses were maintained at 23–25 °C during the thermoneutral period (16 h) of the day. Birds were euthanized via cervical dislocation and necropsied at day 28 post hatch, following 1 week of cyclic heat stress. In terms of bird internal temperatures, heat stress individuals averaged a temperature of 43.5 C while control birds averaged a lower 41 C. Livers were flash frozen in liquid nitrogen, and stored at − 80 °C for further processing.

### RNA and library preparation

As described in the previous studies [7] (Hubbard et al., [2, 7]) (Jastrebski et al., [2]), 45 mg of the left lobe of 8 thermoneutral and 8 heat stress liver samples were homogenized and RNA was extracted using the mirVana miRNA Isolation Kit (Ambion, Austin, TX) as per manufacturer instructions. They were quantified using the Qubit 2.0 Fluorometer (Qubit, New York, NY). Samples were checked for quality using the Fragment Analyzer (Advanced Analytical, Ankeny, IA) at the Delaware Biotechnology Institute (DBI, Newark, DE). Libraries were made using the 50 base pair length reads Illumina TruSeq Stranded mRNA Sample Preparation Kit (Illumina, San Diego, CA) per manufacturer instructions and sent to DBI for sequencing. All reads were mapped to the latest NCBI release of the chicken genome at the time of data collection and accompanying annotation, GalGal4. Mapping was done with Tophat2 and Cufflinks2, with raw counts quantification by featureCounts and differential expression accomplished with edgeR. Differentially expressed genes were identified as those with a *p*-value < .05 using edgeR.

### Metabolome sample preparation

As described in [2, 7] 50 mg of 12 thermoneutral and 11 heat stress liver samples were sent to Metabolon (Durham, NC), for analysis of the metabolome. All of the samples used for the transcriptome analysis were included in the metabolomic sample set. Samples were analyzed as previously described [10]. Samples were prepared using the MicroLab STAR system from Hamilton Company (Reno, NV) using in house recovery standards prior to extraction for QC purposes. Extract was divided into fractions for two reverse phase (RP)/UPLC-MS/MS methods (positive and negative ion mode electrospray ionization), and one for HILIC/UPLC-MS/MS with negative ion mode ESI. Several control were used, including the use of technical replicates, extracted water samples as blanks, and in house QC samples to monitor chromatographic alignment. All UPLC-MS/MS methods used a waters ACQUITY UPLC and Thermo Scientific Q-Exactive high-resolution mass spectrometer. Each sample extract was dried and reconstituted with solvents compatible to each method and solvents included a series of standards at fixed concentrations. Metabolon used hardware and software extract created by the company to extract, peak-identify, and QC process the raw data. Compounds were identified using a Metabolon maintained library of purified standards or recurrent unknown entries. Data is provided as a Additional file 1. A total 527 compounds have been identified and registered in Metabolon’s library and quantified in our dataset. The data was statistically analyzed using a Welch’s two-sample t-test following a log transformation and imputation of missing values with the minimum observed value for each compound. The company provided an analysis that included pathway visualizations. These pathway analyses were then incorporated with the transcriptome data to create a more complete view of changing pathways.

## Results

### Output from K-means, random forest, and subsequent hierarchical clustering

**Fig. 4:**
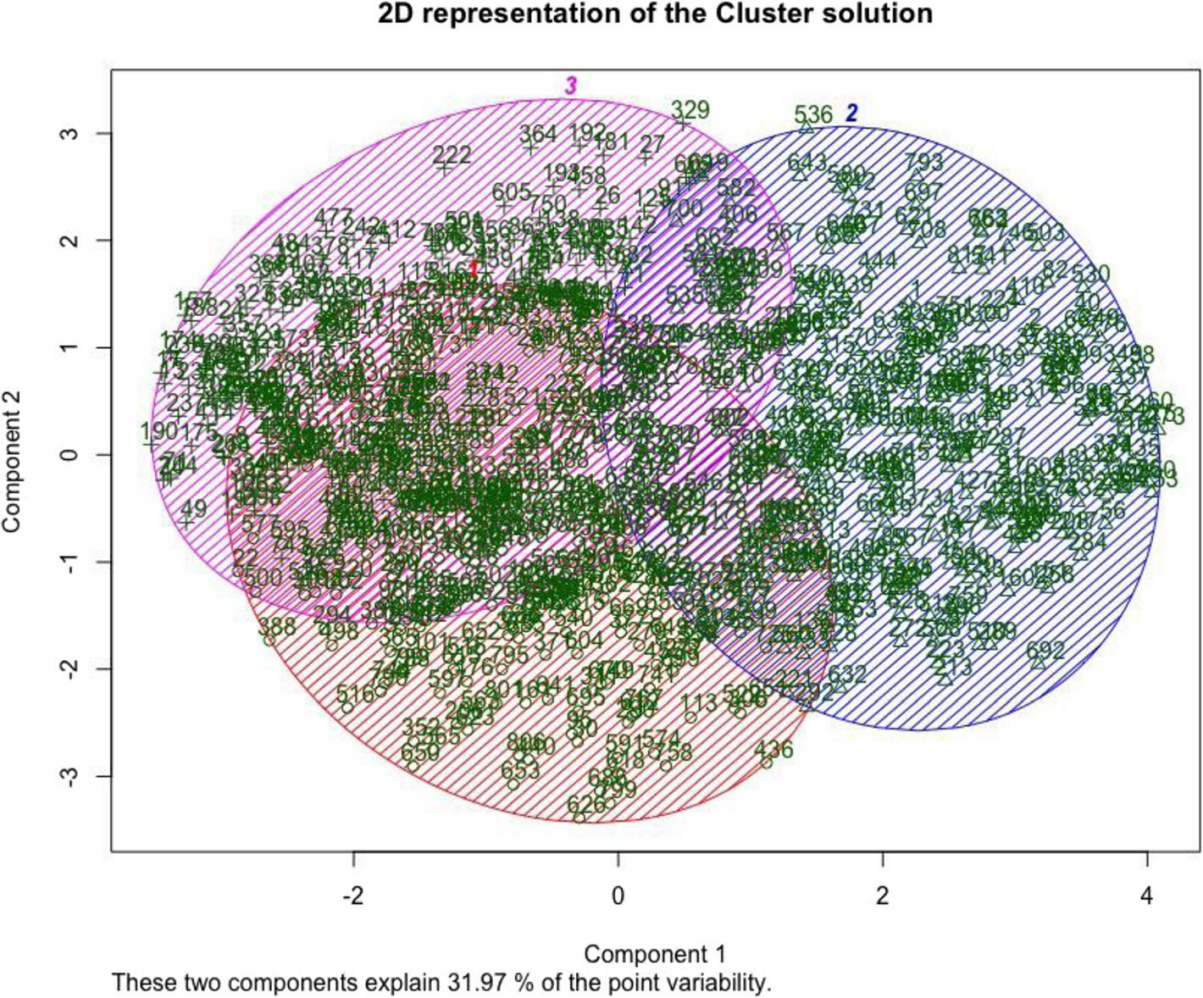
K-means clustering of all compounds. Despite overlap between clusters, these groupings provide an initial separation of biologically relevant groups to prevent overcrowding in subsequent analyses

**Fig. 5:**
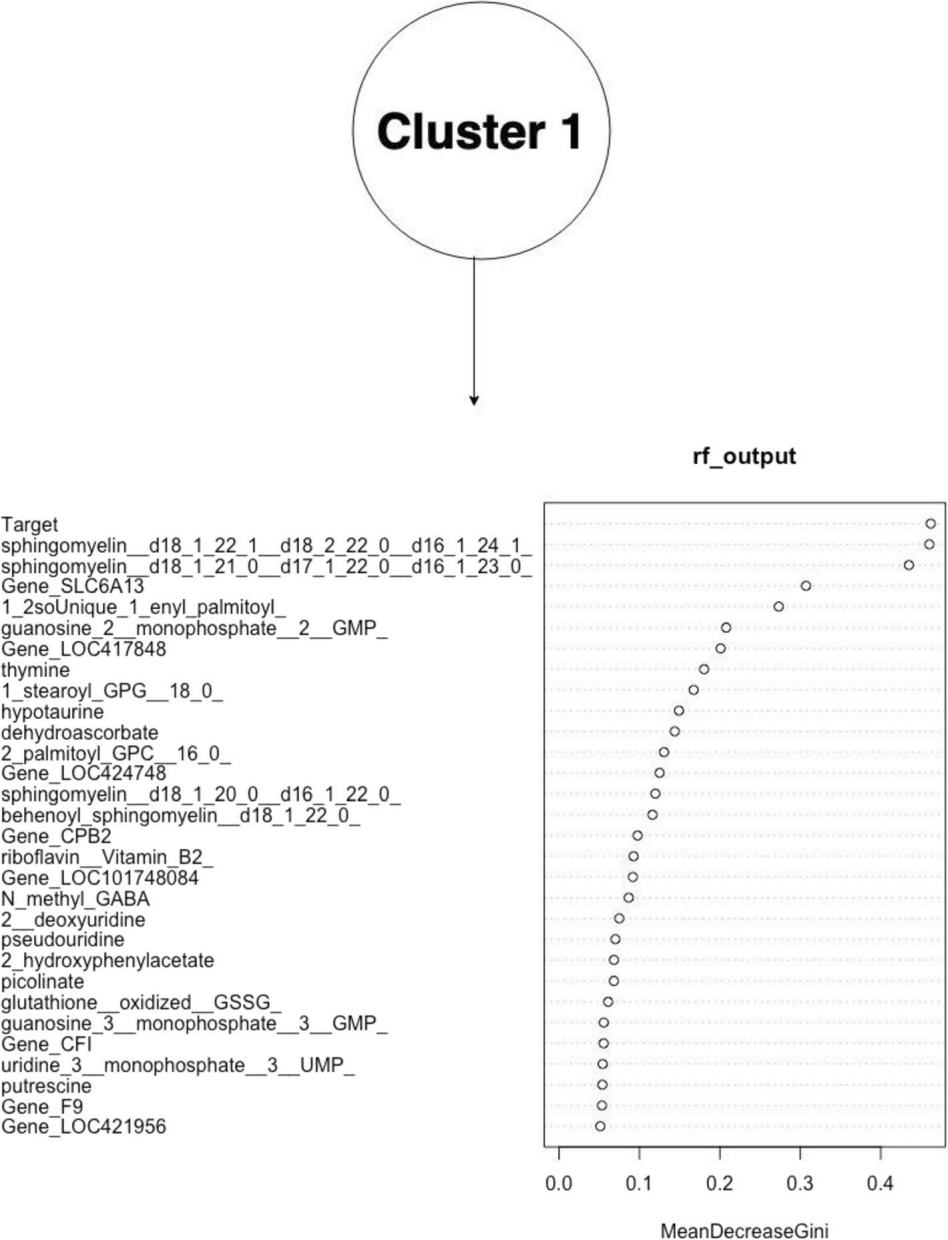
Ranking of top 29 biomolecules in k-means cluster k = 1 prioritized by random forests, by mean improvement in Gini impurity

**Fig. 6:**
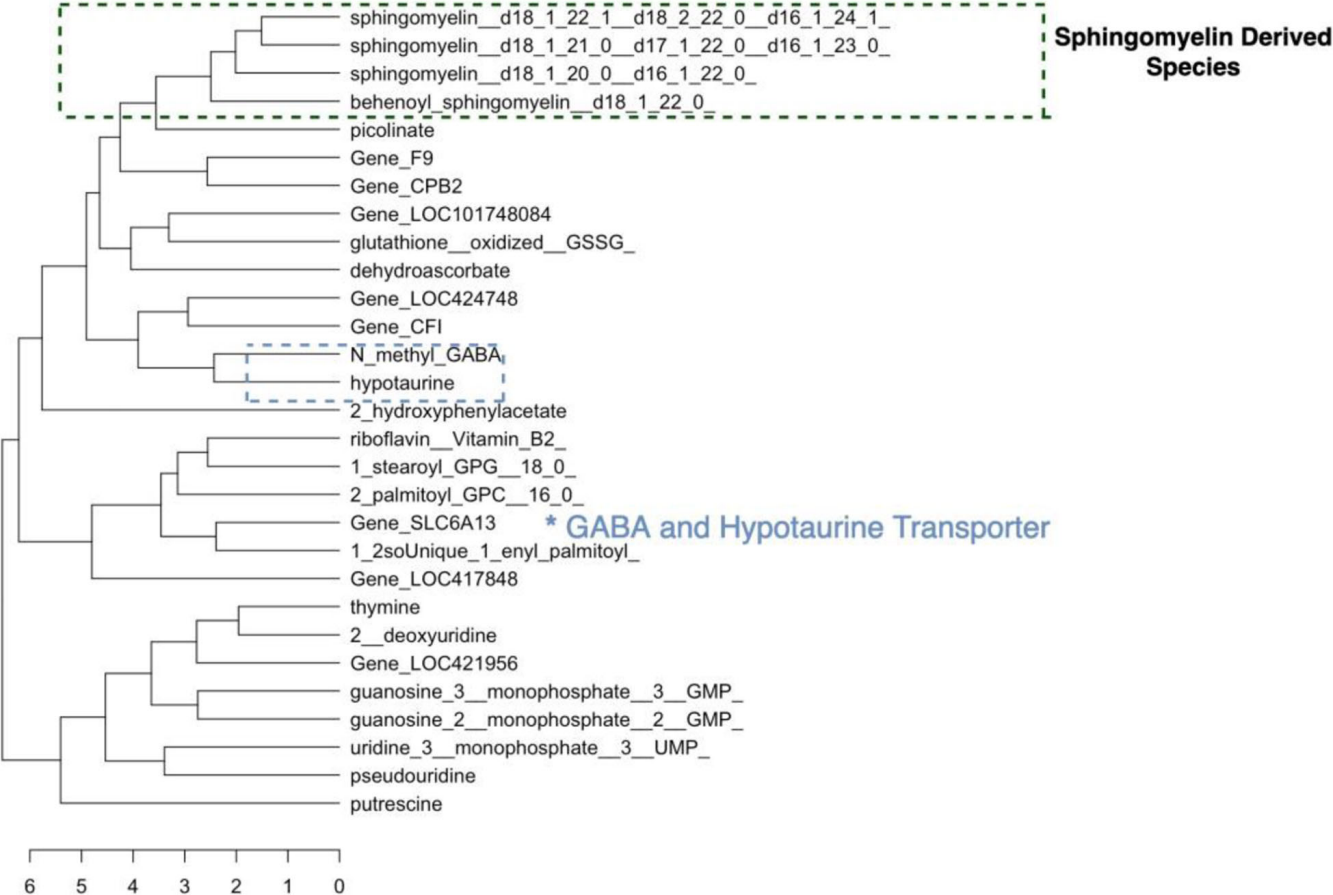
Hierarchical Clustering Cluster 1 Prioritized Biomolecules. Hierarchical clustering dendrogram for prioritized biomolecules in cluster 1

**Fig. 7:**
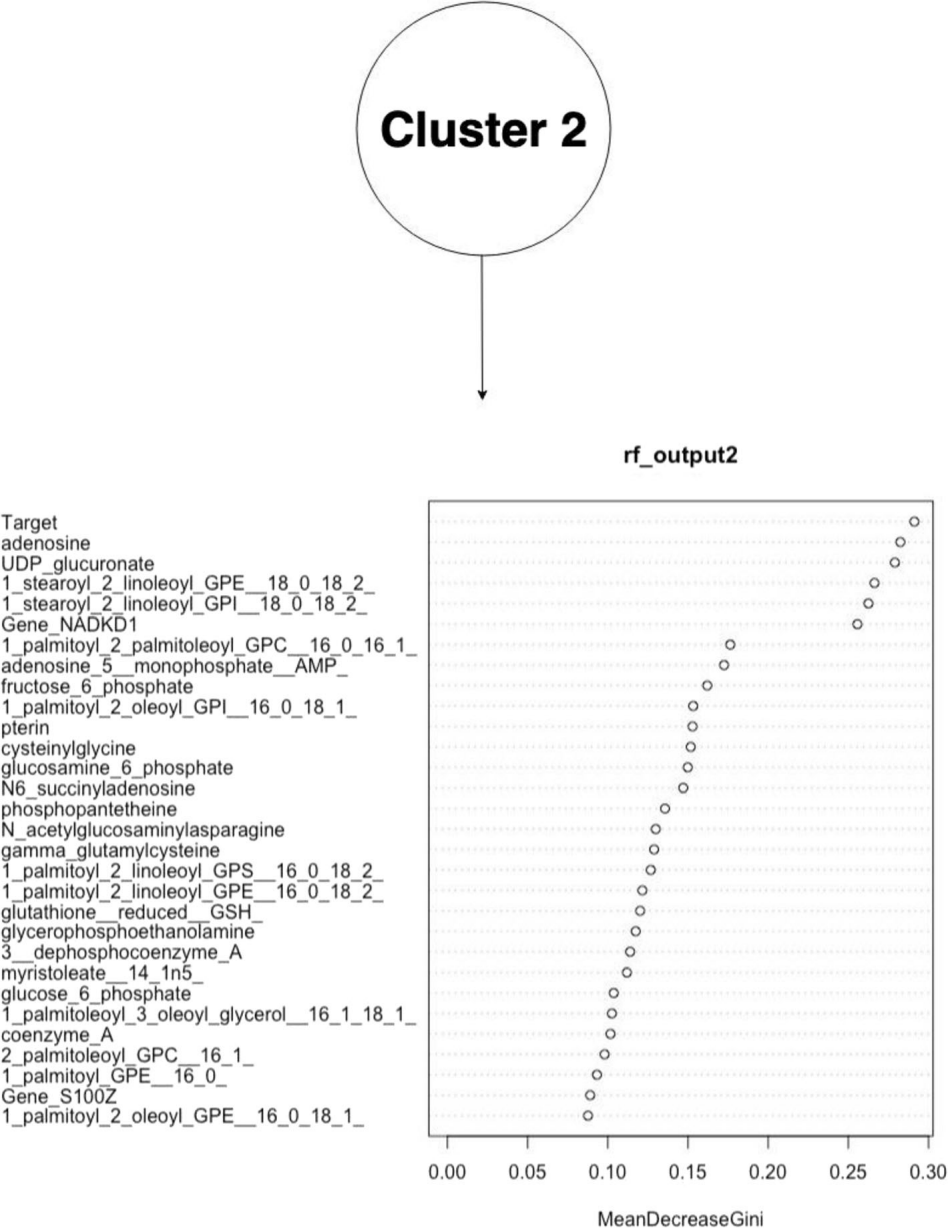
Ranking of top 29 biomolecules in k-means cluster k = 2 prioritized by random forests, by mean improvement in Gini impurity

**Fig. 8:**
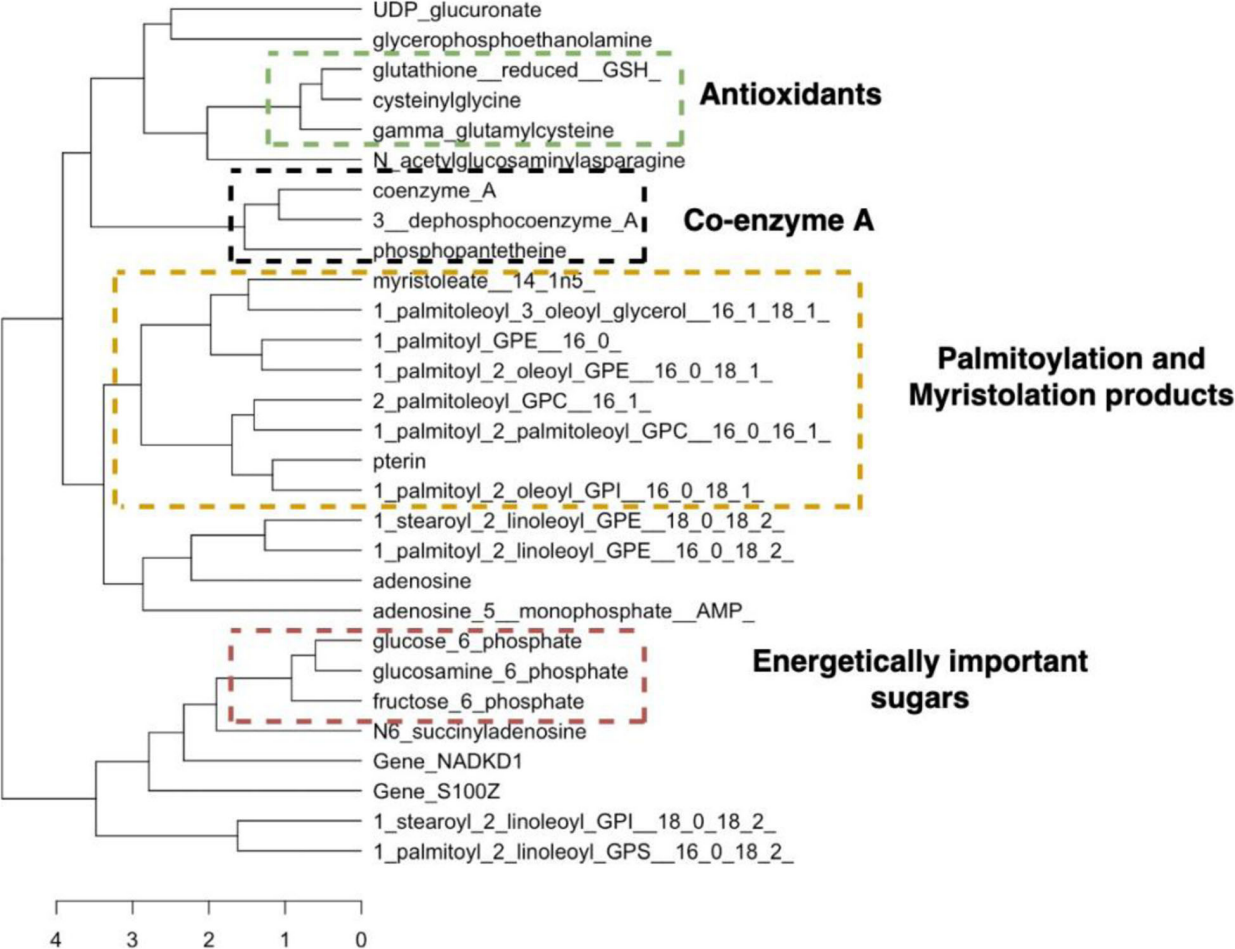
Hierarchical clustering dendrogram for prioritized biomolecules in cluster 2

**Fig. 9:**
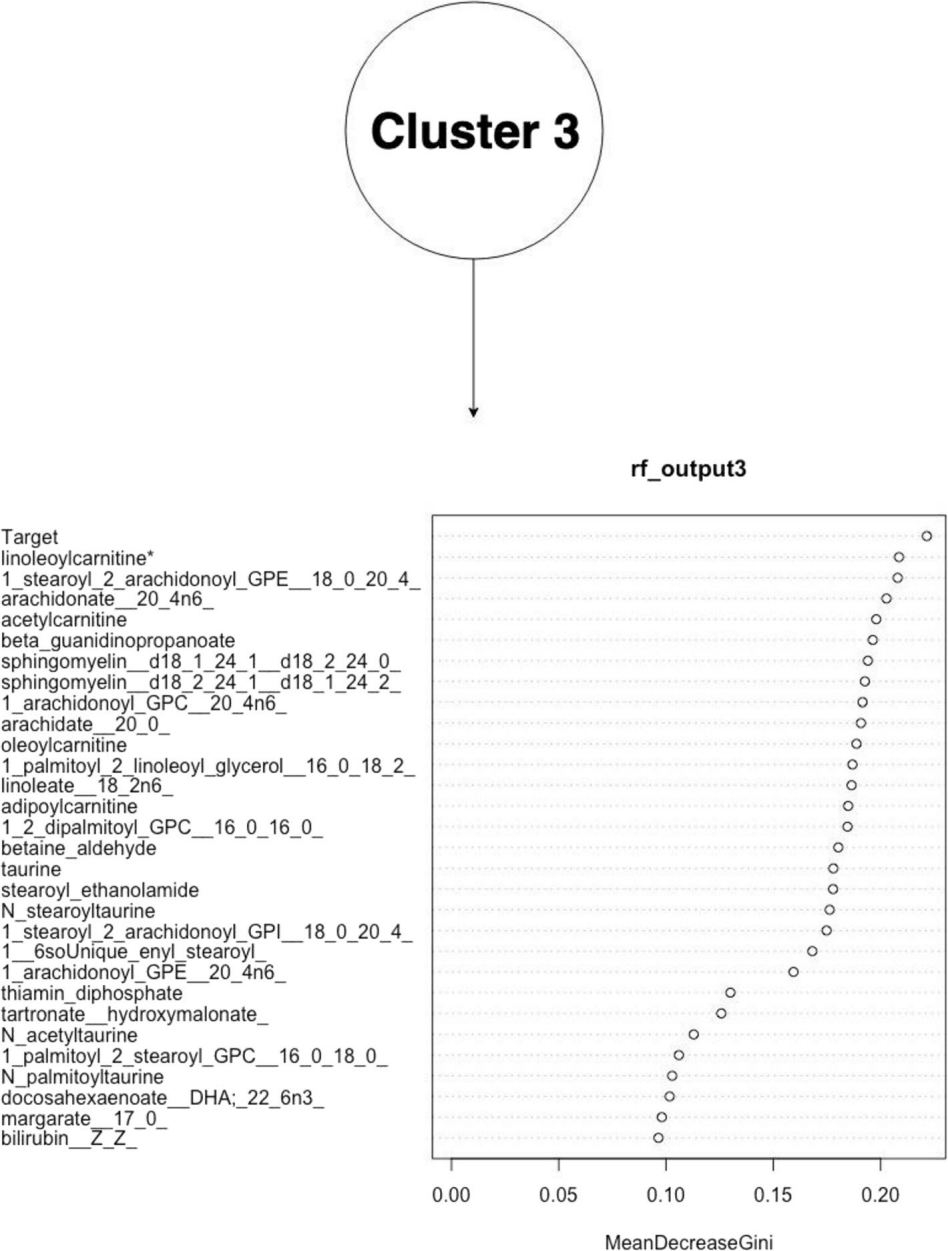
Ranking of top 29 biomolecules in k-means cluster k = 3 prioritized by random forests, by mean improvement in Gini impurity

**Fig. 10:**
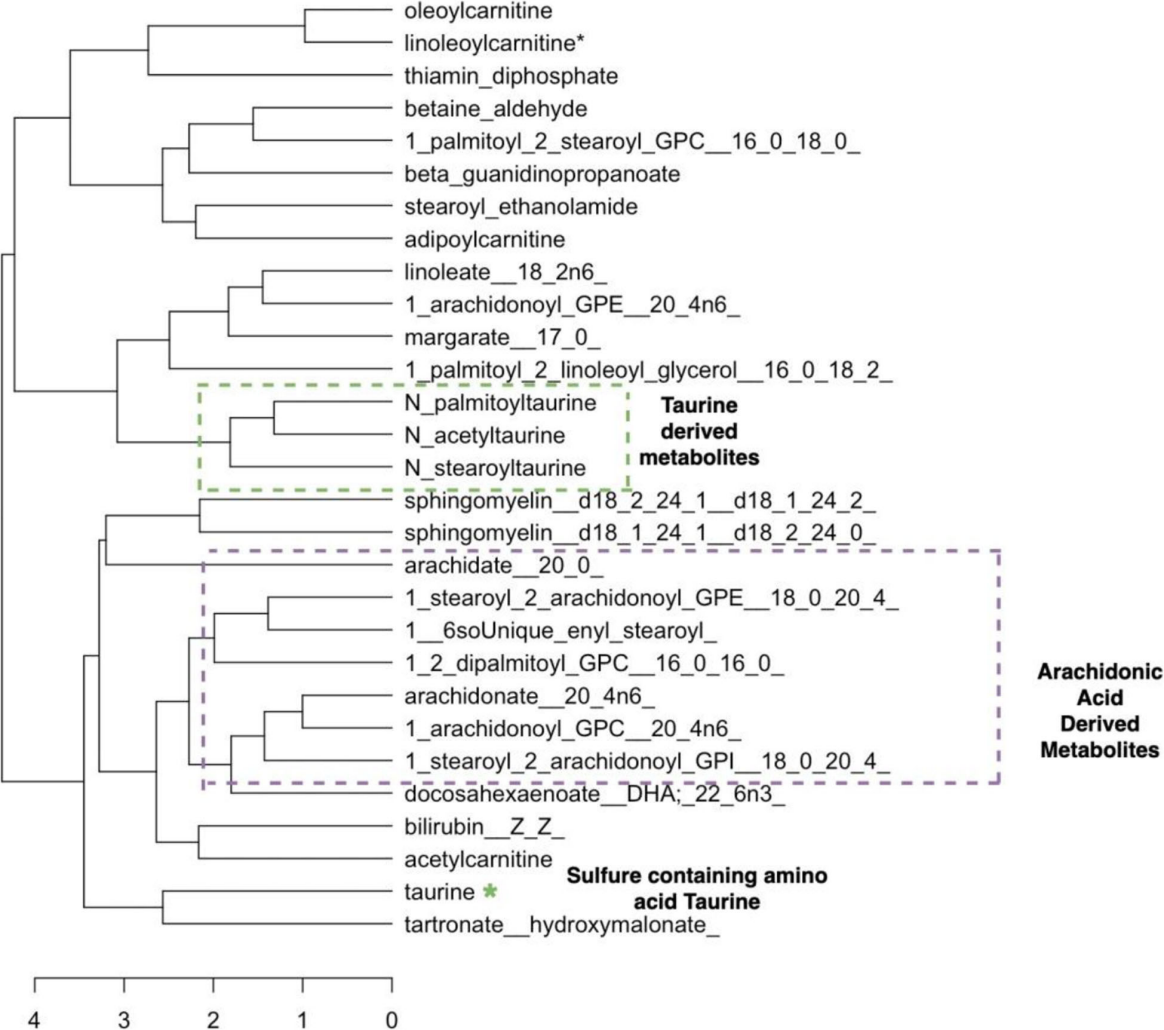
Hierarchical clustering dendrogram for prioritized biomolecules in cluster 3

The figures above (Figs. 4, 5, 6, 7, 8, 9, 10) depict implementations of the statistical procedures as described in the methods (Figs. 1, 2 and 3). Figures 8, 6, 10 are results for hierarchical clustering on the top 29 biomolecules (Figs. 5, 7and 9) from the transcriptome and metabolome ranked by classifying power as determined by random forest, in each of the clusters from k-means. Hierarchical clustering was conducted on these prioritized biomolecules in order to separate out biologically important clusters with similar patterns of measurement across the birds. These biologically meaningful units are highlighted in the Figs. 8, 6, 10.

In each k-means cluster this workflow prioritizes broad groups of biologically related compounds such as sulfur containing compounds related to amino acid metabolism (taurine, hypotaurine, cysteinylglycine) (Figs. 5 and 6), sugars (fructose-6-phosphate, glucose-6-phosphate) (Figs. 7 and 8), lipids (stearoyl ethanolamide, various sphingomyelins) (Figs. 8 and 9) and (Figs. 4, 5, 6). Importantly, elements of these three axes of regulation are spread across the k-means clusters. The arrangement of these clusters in PC-space can be seen in Fig. 4. Cluster 3 contains many biomolecules such as sugars and antioxidants whose levels are increased under heat stress, owing to the orientation of these biomolecules with the first principal component. Clusters 1 and 3, however, contain many lipids and sulfur-containing intermediate species which are lower under heat stress. For example, sphingomyelin species whose levels are lower under heat stress (*p* < .05) are found in clusters 1 and 3 (Fig. 6).

The biomolecules associated with energy production in cluster 2 and which are consistently prioritized by random forests include sugars (fructuose-6-phosphate, glucose-6-phospahte) and anti-oxidant molecules such as reduced glutathione and cysteinylglcine) (Fig. 7). Meanwhile, many of the heat impacted compounds in cluster k = 1 describe products of sulfur metabolism and amino acid catabolism (taurine, hypotaurine, N-stearoyltaurine) whose levels are lower under heat stress (p < .05) (Fig. 9). The first iterative combination of statistical learning approaches (k-means followed by random forests) is effective at separating biologically functional classes of compounds (lipids, sugars and sulfur containing amino acids).

Hierarchical clustering further organizes these biomolecules by relationships between genes and metabolites. This degree of resolution captures regulation across the transcriptome and metabolome. For example, the compounds methyl GABA and hypotaurine in k-means cluster 1 are among the top biomolecules prioritized by random forests (Fig. 5). They subsequently cluster next to one another in hierarchical clustering (Fig. 6). These two compounds are related to the gene SLC6A13 as either substrates (hypotaurine) or derivative of a substrate (N_methyl_GABA) [11]. Mouse knockouts of SLC6A13 are known to have 50% lower taurine levels in the liver compared to wildtype individuals [12]. The downregulated gens SLC6A13 is additionally prioritized by random forests by its expression pattern (Fig. 5). In addition to the relationship between hypotaurine and N_methyl GABA as substrates of the SLC6A13 transporter, hierarchical clustering resolves relationships between derivatives of energy related sugar molecules.

For example, glucose-6-phosphate, fructose-6-phosphate, and glucosamine-6-phosphate cluster together (Fig. 8). These are all compounds prioritized by random forests and found in k-means cluster 2 (Fig. 7). Additionally, an entire trio of co-enzyme A derived compounds (coenzyme A, 3_dephosphocoenzyme A, phospohopantetheine) from this k-means cluster are grouped together under hierarchical clustering (Fig. 8). In addition to its role in the citric acid cycle, Coenzyme A is critical to fatty acid oxidation.

Sulfur species found in clusters 1 and 2 that are catabolic intermediates to anti-oxidant production (hypotaurine, taurine) or lipid derivatives of taurine (N_acetyltaurine, N_palmitoyltaurine, N_stearoyl_taurine) (Fig. 10) and are lower under heat stress and group together under hierarchical clustering in their respective clusters. Sulfur metabolism end-products such as antioxidants in k-means cluster k = 2 glutathione and cysteinylglycine whose levels increase under heat stress, meanwhile, cluster together (Fig. 8). In this cluster of k = 2, a suite of upregulated lipids similarly cluster together, representing general products of myristoylation and palmitoylation (myristoleate-14-1n15 and various palmitoyl-olyeol species) (Fig. 8). The presence of metabolically important palmitoylate and myristoylated lipids in k-means = 2 whose levels are increased by heat stress contrasts with the signaling and structural sphingomyelin lipids in k-means = 2 and k-means = 1 cluster.

**Fig. 11:**
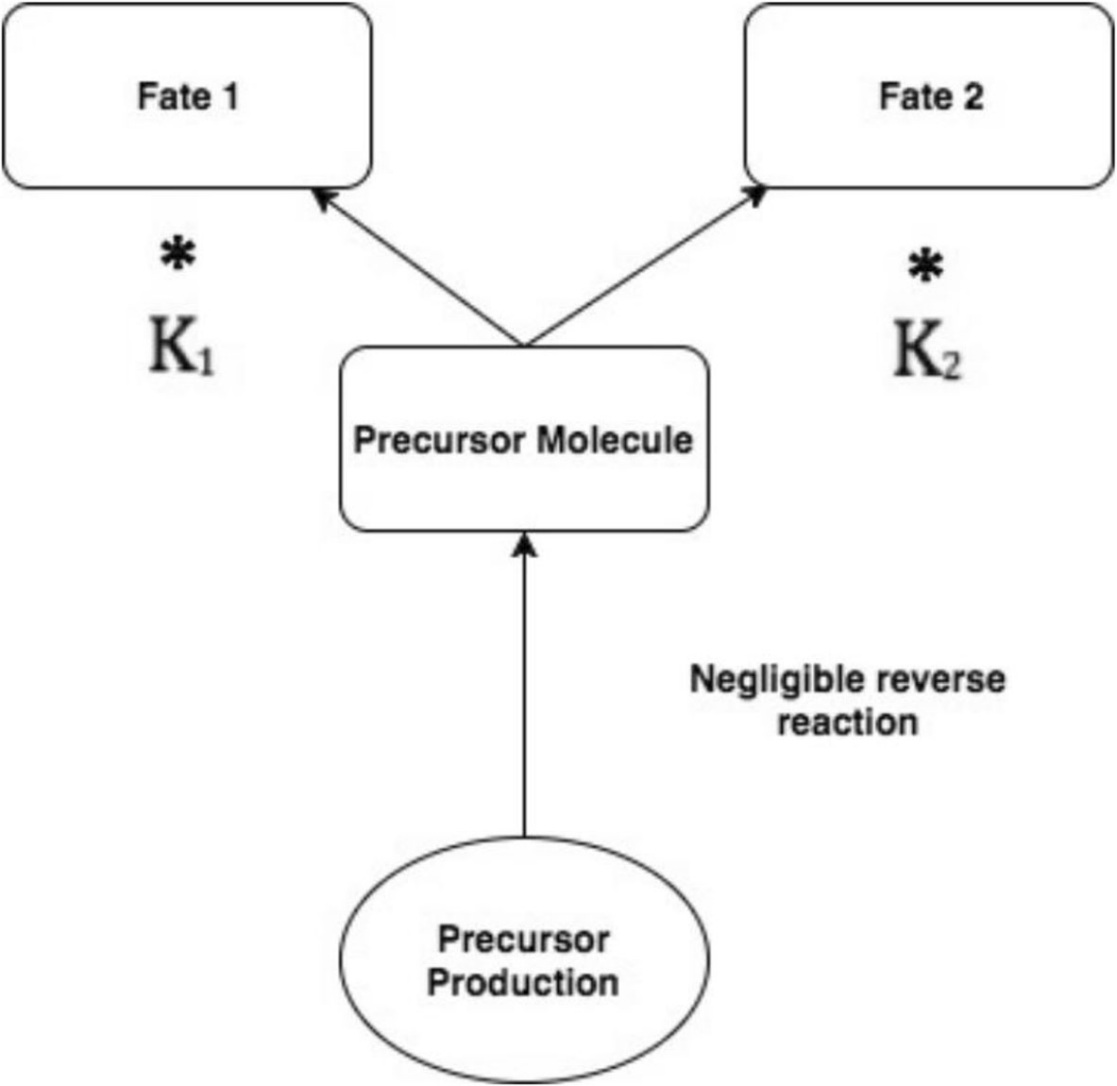
Illustration of the components of a metabolic fork

**Fig. 12:**
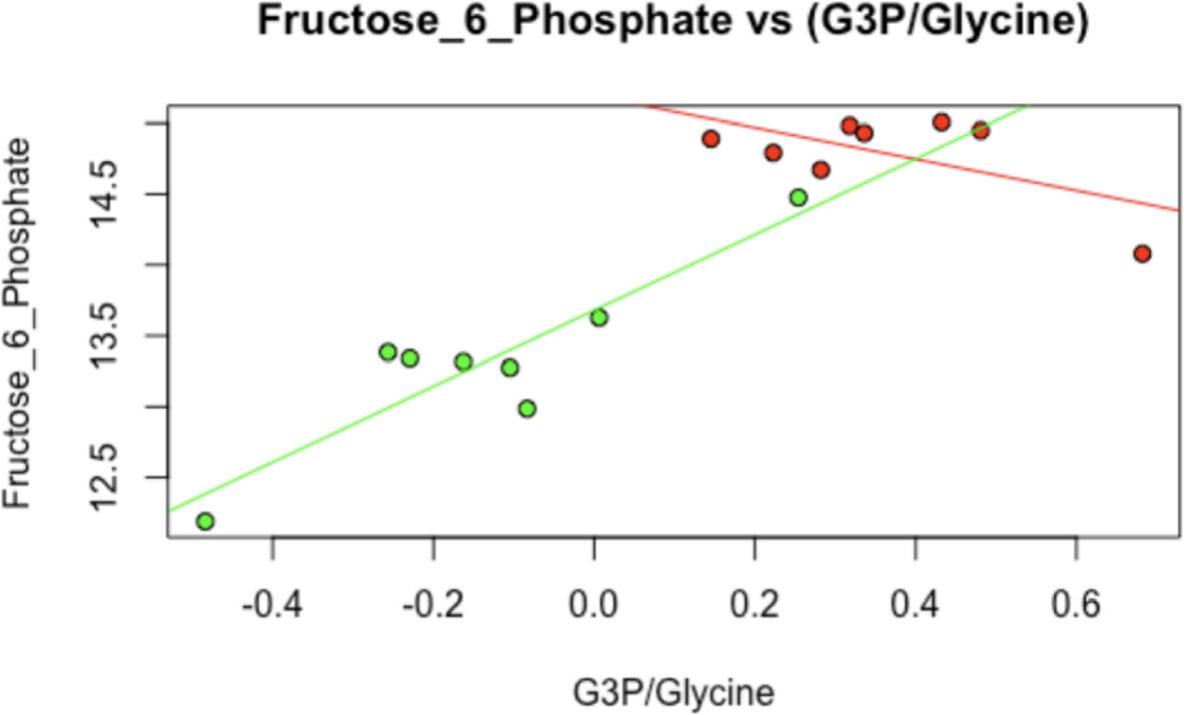
Illustration of the components of a metabolic fork

Consistent with the involvement of multiple biological systems in the heat stress response identified through the statistical learning methods, the model of a potential “metabolic fork” (Fig. 11) described in the model $\mathrm{F}6\mathrm{P}\sim \left(\frac{\mathrm{G}3\mathrm{P}}{\mathrm{glycine}}\right)$ (Fig. 12) represents differential behavior under heat stress (*p*-value of interaction term < .05). This model incorporates elements from lipid metabolism (G3P), sugar metabolism (F6P) and amino acid catabolism (glycine). The model describes a potential regulatory mechanism whereby sugar metabolism is associated with changes in amino acid and lipid metabolism (Additional file 3). The gene FBP2 which encodes a rate-limiting enzyme in gluconeogenesis is upregulated during heat stress (*p*-value < .05).

## Discussion

Our complete analysis, which combines statistical learning techniques with hypothesis-free modeling of metabolite ratios, is able to propose novel hypotheses while recapitulating significant known biology from the liver metabolome and transcriptome (Fig. 1). Importantly, this perspective identifies changes in compounds with roles across organelles that are increasingly thought to have important functions in the heat stress response.

Much interesting biology, for example, relates to changes in the cell membrane. Our pipeline prioritizes widespread shifts in levels of constituent lipids, for example. The exact mechanisms by which these shifts occur remain unclear, but accumulating evidence suggests these changes in the cell membrane exert important downstream effects on heat stress responsive genes and metabolites. Hierarchical clustering identifies groups of these compounds such as the many sphingomyelin species which cluster together in their respective k-means clusters k = 2 and k = 3 (Figs. 6 and 10), suggesting shared regulation of their changes under heat stress. At least some of these may be driven by dietary changes that result from heat stress such as decreased feed consumption. Linoleic acid levels are lower under heat stress, for example, and the compound must be acquired by diet., Linoleic acids is a precursor to arachidonic acid and the latter emerges as a strong heat stress associated biomolecule and whose detected levels are lower under heat stress. Downstream arachidonic acid derivatives are similarly decreased, many of which have roles in inflammatory response. Several arachidonic acid derivatives prioritized by random forests from k-means cluster k = 3 group together under hierarchical clustering, consistent with shared upstream regulation, possibly through linoleic acid. These compounds are highlighted in Fig. 10 and include arachidonate and various stearoyl arachidonate among other compounds.

Other biomolecules prioritized by random forests and which cluster together under hierarchical clustering include additional lipids related to signaling and fatty acid oxidation – such as adipoylcarnitine and the taurine related endocannabinoids N-oleoy N-Stearoyl taurine (Figs. 9 and 10). These compounds, derived from taurine, thus represent a possible intersection between signaling lipids and sulfur metabolism via coupling with taurine. All of these compounds occur at lower concentrations under heat stress. The similarities in their metabolic profiles is supported by the adjacent clustering of N-palmitoyltaurine, N-acetyltaurine and N-stearoyltaurine the dendrogram (Fig. 10). While the specific mechanisms of their regulation remain an area of active research, lipid changes are increasingly recognized as potential regulators of heat stress at a fundamental level [13] .

Recent studies have focused on nuances of the heat stress response by revising the model that it is primarily triggered by the presence of unfolded proteins [14]. For example, lipids in the cell membrane may detect membrane disorder and other physical consequences of heat stress and trigger signal cascades [13]. The evolutionary value of using a thermo-sensitive organelle such as the cell membrane to refine the heat stress response lies in the advantage of being able to regulate homeostasis through sensitive adjustments that have meaningful influences on cell fate [15]. Many compounds prioritized by our pipeline are lipids that may be involved in such processes at a cellular level, and consequently influence bird metabolism.

### Heat stress, membranes and lipids

The sophisticated signaling environment created by the cell membrane is comprised of a diverse set of lipids and proteins. Among these is an abundance of sphingolipids that form rafts in the membrane and possess important signaling roles [15]. The organization of the cell membrane is intricate and becomes dynamic under stress response. Important structural changes occur through interactions with membrane proteins, the gating of which possess thermal sensitivity [16] Additionally, heat causes changes in physical attributes such as diffusion and dimerization rates. Measurements suggest these characteristics change in a predictable fashion during even mild heat stress events [16]. Thus, the cell membrane is well equipped to sense relative temperature changes.

Not surprisingly, among the compounds prioritized by our pipeline are many lipids with a diverse set of signaling and structural roles. During episodes of heat stress, mechanisms to endure temperature shifts focus generally on maintaining the integrity of cellular processes and such pathways can be causally regulated by changes in cell membrane disorder [17] . For example, regulation of heat shock factors can be influenced by addition of saturated and unsaturated fatty acids, with the former inducing expression and the latter suppressing it [18].

The possibility that the qualities of the cellular membrane make it an ideal substrate in which to store ‘memory’ or serve as a ‘control center’ for a physiological response in terms of the composition of density and sensing molecules is extremely interesting biologically. This could prove extremely important in terms of identifying the most upstream mechanistic regulators of the overall response. Indeed, changes in membrane fluidity induced via alcohols triggers systemic responses paralleling those caused by heat stress, albeit in the absence of any thermal activation. Such changes include hyperpolarization of the mitochondrial membrane [19]. Such experimental work confirms the role of lipids from a regulatory perspective and the influence of the heat stress response across organelles.

Among the cell membrane lipids influenced by heat stress and which are prioritized among their respective clusters is a number of sphingomyelin species (Figs. 5 and 6). These are substantially down regulated under heat stress and emerge as strong classifiers in clusters one and three. Importantly, these compounds are broadly similar to one another under hierarchical clustering (Fig. 5). This is an interesting observation in the context that sphingolipids are up-regulated in the early phases of acute heat stress in studies of yeast [20] . Many of these sphingomyelin species group together under hierarchical clustering along with suppressed inflammatory arachidonic acid derivatives (Fig. 10). Their general attenuation may be an important aspect of physiological adaptation to the long term heat stress experienced by the birds, with the pattern of variance in their levels indicative of bird acclimatization.

### Anti-oxidants and energy burden

Heat stress entails a number of challenges that endanger cell function and which must be addressed in order to preserve homeostasis. The management and deployment of downstream protective systems such as antioxidants can be quite independent from the initial sensory capacity of the cell membrane and its heat sensing pathways. These changes, for example, must mitigate cellular damage that could result from ongoing heat stress. Such pathways are essential to the heat stress response, as they manage of general consequences of oxidative damage. Several precursors of anti-oxidants, as well as such compounds themselves, are identified as strong classifiers of heat stress treatment within each k-means cluster. These compounds, such as glutathione and its derivative cysteinylglycine (Fig. 8), manage the effects of toxic intermediates resulting from increased energy production, mitigating their ability to damage DNA or organelles. Their production may exploit the carbon backbones of amino acids released by catabolized protein. The importance of tight coupling between sulfur and antioxidant metabolism is supported by the close grouping of various sulfur derivatives (reduced gluthathione, cysteinylglycine, gamma-glutamylcysteine) under hierarchical clustering (Fig. 8).

Not surprisingly, given the relationship between oxidation and energy production, some of these biomolecules are associated with changes in mitochondrial activity. Even slight changes in cell resting state can have dramatic changes on the production of reactive oxygen species and the behavior of the mitochondria [21]. Molecules associated with mitochondrial performance are computationally recognized as potential biomolecules of the heat stress response. This suggests that mitochondrial conditions are closely related to heat stress in general, and that the cell adjusts antioxidant levels accordingly.

At the same time that sugars and other energy-related metabolites show upregulation, an important class of lipids involved in the carnitine shuttle system that transports fatty acids to the mitochondria shows consistent downregulation. These carnitine species (stearoylcarnitine, adipoylcarnitine) are identified as strong heat stress associated biomolecules among their clusters and group tightly under hierarchical clustering (Fig. 6). Such patterns suggest sweeping downregulation of fatty acid oxidation pathways, as metabolism is increasingly driven by gluconeogenesis. Transcriptome changes in heat stress have been established as supporting a coordinated shift in lipid and sugar management [2].

Genes that emerge from the k-means cluster containing gluconeogenesis biomolecules include NAD kinase (NADKD1) and S100 Calcium Binding Protein Z (S100Z) These genes cluster next to one another, while also close to core upregulated gluconeogenesis compounds F6P and G6P. NADKD1 is a Nicotinamide Adenine Dinucleotide (NAD) kinase responsible for Nicotinamide Adenine Dinucleotide Phosphate.

(NADP) production, while S100Z is a calcium binding protein. Calcium released.

During oxidative stress can trigger cell death [22]. Thus,

upregulated S100Z may be important to mitigating apoptosis.

NADKD1, however, may play a role in lipid metabolism, by producing NADP that will be reduced to NADH by the pentose phosphate pathway and thus providing reducing power for lipid production [23]. Thus, NADKD1 production provides a potential link between gluconeogenesis and lipid production, at the same time lipid oxidation is decreased. The shift away from lipid oxidation is consistent with increases in coenzyme A.

The shift towards gluconeogenesis is supported strongly from a mechanistic standpoint by the metabolic fork (Fig. 11). The metabolic fork provides evidence of large-scale redirection of carbon resources released from the catabolized glycine. to complement purely correlation-based strategies with mechanistic hypotheses.

### Metabolic forks resulting from gene regulation

One of the top differentially regulated triplets contains two compounds prioritized through hierarchical clustering on top biomolecules on a k-means cluster. This is consistent with gene important expression changes, such as those involving FBP2. The three members of the triplet span gluconeogenesis (fructose-6-phosphate), glyceroneogenesis (glycerol-3-phosphate) and amino acid catabolism (glycine). Pairwise correlations between each node are provided on the corresponding edge. A proposed mechanism for the observed pattern is that catabolized glycine is preferentially shunted towards gluconeogenesis under heat stress, thus contributing to F6P production. Increasingly fueled by carbon backbones provided by amino acids from catabolized proteins, gluconeogenesis decouples from glyceroneogenesis under heat stress.

The ratio of G3P to glycine represents the tendency of catabolized amino acids to become backbones for fats, as opposed to sugars. This changes as a function of increased demand for sugar under heat stress and is corroborated by increase in the gene Fructose-Bisphosphatase-2 (FBP2) encoding the rate-limiting gene for gluconeogenesis.

## Conclusions

Interest in the heat stress response is broad, stretching from plant physiology to human clinical research, with insights potentially applicable across taxa due to the deep conservation of cell signaling pathways. Next generation sequencing technologies provide new experimental perspectives from which to explore such systems. During the past several years, the advent of next generation sequencing tools has produced a deluge of data. However, methods to process that data have been lacking. Combining the information from transcriptome and metabolite data, and multi organ datasets compounds this challenge. The capacity to link patterns of heterogeneity to pathway importance is an approach that can ease the burden of prioritizing compounds in such a setting. Here, we do so and leverage a combination of relative tissue enrichment and statistical learning approaches to prioritize compounds based on their ability to identify samples as belonging to heat stress or control conditions. We demonstrate signatures of the heat stress response across several important systems. Importantly, this is a very general strategy that works with any type of continuous data, rendering it applicable to both metabolome and transcriptome data and flexible enough to accommodate future “-omics” data.

While recapitulating known biology, our analysis also proposes new hypotheses about heat stress regulation that relates to systems controlled by a diverse range of organelles. These can be explored through future experimentation. Additionally, the metabolic fingerprint of heat stress provides candidates for feed supplementation studies. Thus, this study proposes a general workflow to integrate high dimensional, complex datasets in order to yield testable hypotheses about biology.

## Additional files

Additional file 1: Appendices_Supplementary_EdgeR_Data.xlsx. -*p*-values for all genes using the statistical program edgeR. (TXT 1210 kb)

Additional file 2: Appendices_Supplementary_Metabolomics_Data.csv. -raw metabolomics data utilized in the study (CSV 78 kb)

Additional file 3: F6P_G3P_Glycine_triplet_BMC. R Code for the linear model used to describe the metabolic fork (R 1 kb)

Additional file 4: liver_enriched_genes_for_manuscript.txt. List of liver enriched genes. (TXT 3 kb)

## Data Availability

Transcriptome sequencing data is publicly available through GEO series accession number GSE95088 (https://www.ncbi.nlm.nih.gov/geo/query/acc.cgi?acc=GSE95088). Metabolome data is included as Additional file 2.

## Abbreviations

F6P: Fructose-6-phosphate
FBP2: Fructose-Bisphosphatase-2
G3P: Glycerol-3-phosphate
GTEX: Genotype Tissue Expression
GWA: Genome Wide Association
K1: Rate constant for forward reaction
K2: Rate constant for reverse reaction
NAD: Nicotinamide Adenine Dinucleotide
NADKD1: NAD Kinase, mitochondrial
NADP: Nicotinamide Adenine Dinucleotide Phosphate
NADPH: Nicotinamide Adenine Dinucleotide Phosphate, Reduced
RNA-seq: Ribonucleic Acid Sequencing
S100Z: S100 Calcium Binding Protein Z
SNP: Single Nucleotide Polymorphism
